# Overweight and obesity and their associated factors among early adolescence school children in urban and rural Portugal

**DOI:** 10.1186/s40795-017-0134-6

**Published:** 2017-02-20

**Authors:** Luísa Maria de Morais Macieira, Jorge Manuel Tavares Lopes de Andrade Saraiva, Lélita da Conceição Santos

**Affiliations:** 1 University Paediatric Clinic, Faculty of Medicine, University of Coimbra, Av. Afonso Romão, 3000-602 Coimbra, Portugal; 2 Dietetics and Nutrition at the College of Health Technology, Av. Afonso Romão, 3000-602 Coimbra, Portugal; 3 Medical Genetics Unit, Paediatric Hospital, Centro Hospitalar e Universitário de Coimbra, Rua Afrânio Peixoto n° 28, 3000-013 Coimbra, Portugal; 4 Internal Medicine Department, Centro Hospitalar e Universitário de Coimbra, Rua Afrânio Peixoto n° 28, 3000-013 Coimbra, Portugal; 5 Faculty of Medicine, University of Coimbra, Av. Afonso Romão, 3000-602 Coimbra, Portugal

**Keywords:** Obesity, Overweight, Child, Adolescent, Eating habits, Sedentary lifestyle

## Abstract

**Background:**

Obesity is defined as an abnormal or excessive accumulation of body fat and it is currently one of the most concerning public health issues, as it is related to a wide range of serious diseases and disorders. The study of the causes of obesity is multifactorial, and its diagnosis requires specific methods. Its management is complex, and it is crucial that it is handled appropriately, and its primary focus should be on prevention through lifestyle changes.

The objectives of this study are to determine the prevalence of overweight/obesity in adolescents of both genders, aged 10 to 12 years, from different geographical environments (rural and urban), as well as to identify the underlying risk factors related to the respective obesity rates, namely: family environment, eating habits, and physical exercise.

**Methods:**

An observational, cross-sectional study in a sample of 129 adolescents aged 10 to 12 years was conducted. Participants of both genders from rural and urban environments were included in this study. A questionnaire was completed on eating habits and physical activity, focusing on the number of daily meals, meal composition, and sedentary lifestyle habits. An anthropometric assessment was also performed, including weight, height, skinfolds, waist circumference, arm circumference, and percentage of lean mass and body fat, using bioelectrical impedance analysis.

**Results:**

In the rural environment, the obesity rate was 16.9%, with 26.8% being either overweight or obese; whereas in the urban environment, these rates were respectively 16.7% and 33.4%. Living in a rural environment was not an independent predictor of being overweight or obesity, *p* = 0.581, or for increased percentage of body fat, *p* = 0.790. In contrast, being 12 years old, eating high-calorie foods four times a week or less, and having at least one obese parent were predictors of being overweight or obesity. Being 12 years old was also a predictor of gaining moderate to high body fat.

**Conclusions:**

Adolescents’ residence in a rural or urban environment does not affect the occurrence of being overweight, obesity or high body fat. Paternal obesity was an important predictor of obesity in children. Obese fathers tended to serve higher calorie meals to their children.

**Electronic supplementary material:**

The online version of this article (doi:10.1186/s40795-017-0134-6) contains supplementary material, which is available to authorized users.

## Background

According to the World Health Organization (WHO), obesity is defined as “an excessive deposit of body fat (BF) which may result in adverse metabolic consequences, may impair short and long term physical health and create psychological disorders which should not be overlooked” [1, 2]. Obesity is one of the most concerning public health problems of contemporary society; it is multifactorial in origin (including genetics and family environment, as well as being related to cultural, eating, and sedentary habits). Furthermore, it is associated with various diseases or disorders (dyslipidemia, high blood pressure, type 2 diabetes, orthopaedic disorders, hepatic steatosis and psychological disorders). According to the WHO, the incidence of childhood obesity in Europe has risen from 10% to 40% in the last decade, and to over 30% in Portugal [3–5].

Defining and diagnosing obesity in children is challenging. Until a few years ago, obesity was defined as an excessive body weight in relation to height. Currently, it is known that during growth, BF and body mass index (BMI), calculated as weight in kilograms divided by height in metres squared, depend on a number of factors and change according to age. It is also known that during childhood, BMI is strongly correlated with the amount of total BF, making it a more sensitive tool for diagnosing overweight/obesity (OWOB) [6–9].

There are various methods available for assessing body composition: necropsy (highly sensitive), extrapolation, anthropometric parameters, bioelectrical impedance (BIO), densitometry (which measures total BF and its distribution), lean and bone mass, computerised axial tomography, and magnetic resonance imaging.

There are two types of paediatric obesity: primary and secondary. In the case of primary (or exogenous) obesity, there is no apparent underlying cause.

Paediatric primary obesity is a multifactorial aetiological condition which is associated with metabolic, genetic, nutritional, socioeconomic, cultural, psychological, and lifestyle factors. The assessment of paediatric obese patients should focus on a thorough examination of clinical history, a physical examination and, if necessary, a laboratory workup.

The management of paediatric obesity is centred on lifestyle changes, rarely requiring pharmacological or surgical interventions. It is important to follow a set of guidelines, which require a joint effort involving the patient, family, school, healthcare centres, and local institutions, with the latter providing sports and leisure activities. Behavioural therapy is another option, which aims to change family lifestyle habits. If there are associated diseases or disorders, or in severe cases of obesity, pharmacological or surgical therapy may also be required.

To prevent paediatric obesity and related diseases, health education is fundamental. Issues such as the promotion of breastfeeding, healthy eating habits, and physical activity from kindergarten through to adulthood should also be addressed. Knowledge of these issues should be available to politicians, families, and care providers alike, in order to implement a shift in attitudes regarding health promotion and, ultimately, obesity prevention.

There are few studies available that compare adolescent obesity in rural and urban environments [10, 11], and those that do exist are all outside Portugal. To the best of our knowledge, this is the first study to compare the eating habits, physical activity, sedentary habits, and parental BMI of Portuguese adolescents in two different environments (rural and urban). With the present study, we intend to narrow the gap in the literature of studies that compare rural and urban environments with regards to being overweight and/or obesity in adolescents.

The objectives of this study are to determine the prevalence of overweight/obesity in adolescents of both genders, aged 10 to 12 years, from different geographical environments (rural and urban), as well as to identify the underlying risk factors related to the respective obesity rates, namely: family environment, eating habits, sedentary activities and physical exercise.

## Methods

In this study, 129 adolescents of both genders between the ages of 10 and 12 years from two locations in Portugal were included: a rural area (living in and attending a public school in the town of Vila de Rei, Castelo Branco district), and an urban area (living in the centre of the city of Coimbra and attending a private school called *Colégio de S. José* – St. Joseph’s School in Coimbra; this school works within an agreement with the Portuguese Education Ministry, which means that it receives public funding which enables less-well off urban children to attend in this school - Table 1). The selection of the urban school was related to the proximity of the location with the Paediatric Hospital of Coimbra, and to the fact that the majority of the students’ parents having high academic degrees and a high standard of living, whereas the rural school was selected because it was located in a setting with low academic achievement, with a low standard of living, where the majority of students’ parents are engaged in general labour, and it also has one of the lowest birth rates in the country. The exclusion criteria included the following: children whose parents did not sign the consent form; those who had secondary obesity; those receiving chronic pharmacological therapy; those who were lost during follow-up; and those that attended the selected schools but did not live in the respective location (Vila de Rei or Coimbra city). The study was approved by Coimbra’s Paediatric Hospital Ethics Committee (no. 1022, March 31, 2006) and the Medical School Ethics Committee of the University of Coimbra (no. G/295, December 5, 2006). The parents received written information about the study and its objectives, and an informed consent form was signed, and a questionnaire regarding eating and sedentary habits (Additional file 1) was then distributed. The internal consistency of the questionnaire was assessed by Cronbach's alpha, α = 0.59.

**Table 1:** Urban versus rural setting: baseline characteristics

Parameter		Urban (*n* = 57)	Rural (*n* = 72)	Total (*n* = 129)	*p*
Female		42 (73.7%)	33 (45.8%)	75 (58.1%)	0.001
Age (years)	10	14 (24.6%)	34 (47.2%)	48 (37.2%)	0.002
	11	11 (19.3%)	19 (26.4%)	30 (23.3%)	
	12	32 (56.1%)	19 (26.4%)	51 (39.5%)	
Year (School)	4	1 (1.8%)	12 (16.7%)	13 (10.1%)	0.036
	5	19 (33.3%)	22 (30.6%)	41 (31.8%)	
	6	18 (31.6%)	20 (27.8%)	38 (29.5%)	
	7	19 (33.3%)	16 (22.2%)	35 (27.1%)	
	8	0 (0.0%)	2 (2.8%)	2 (1.6%)	

The first section of the questionnaire was aimed at quantifying the number of meals and their composition. The second section addressed sedentary activities, such as time spent watching television, being on a computer, playing videogames, or doing other sedentary activities. It also focused on physical activity outside school physical education, including time spent on these activities in hours per week.

- Clinical assessment

To conduct the anthropometric assessment, internationally recommended methods were used [12]. Anthropometric data were used to categorize students as small, adequate, or large for their gestational age when their birthweight was ≤2,500 g, >2,500 g and ≤4,000 g, and >4,000 g, respectively [13]. These measures were obtained from Individual Health File records.

Waist circumference (WC) was measured, using the midpoint between the costal margin and the iliac crest as a reference. The values were then plotted on the respective percentile curve [14]. Arm circumference (AC) assessments were performed on the non-dominant limb. Three measurements were carried out, and their mean value was used. At a later stage, the AC percentile was also determined [15].

The skinfold thicknesses of the two layers of skin, as well as the subcutaneous fat at a specific point are directly related to total BF [16]. Both the tricep skinfold (TSF) (back of the upper arm, midpoint between the olecranon and the acromion) and the subscapular skinfold (SSF) (between the inferior angle of the scapula and the spine) were also assessed. The final value is presented as the mean of the three measurements and the respective percentile[15].

Arm muscle circumference (AMC) assesses lean mass and this was calculated by measuring the AC, as well as the skinfold at the same point, the bone values were considering as being negligible, using the formula AMC (cm) = AC (cm) – TSF (cm) × 3.1416. The following results were considered normal according to gender: 16.65 for girls and 21.98 for boys. Deviations were calculated using the following formula: % deviation = Calculated value/Normal value × 100. Those with a % deviation between 90% and 95% were considered to have mild depletion, those with deviations between 60% and 90% had moderate depletion, and severe depletion was represented by values under 60%.

The nutritional status of the children was evaluated by taking into account their BMI and they were classified as overweight or pre-obese (BMI: P85-P95), obese (BMI > P95), or morbidly obese (BMI > P97). The BMI of the parents was also determined and was classified as normal weight (BMI: 18-24.9 kg/m^2^), overweight (BMI: 25-29.9 kg/m^2^), and obese (BMI > = 30 kg/m^2^).

Body fat mass was determined by BIO, using the *Tanita TBF 300* model, with reference values for the paediatric population incorporated into the software programme.

b) Statistical analysis

Categorical and numerical variables were characterized by determining the absolute and relative frequencies of the former, and the means and standard deviations of the latter. Comparative analyses were carried out in relation to demographic variables, the results of the eating habits and leisure activities questionnaire, clinical assessments, and biological parameters. Comparisons between both groups with regard to the categorical variables were conducted using the Chi-Square Test, or Fisher’s Exact Test. Regarding the continuous variables, T-Tests were used to compare the means whenever possible, otherwise, the Mann-Whitney U Test was used to compare the medians.

Predictors of the existence of OWOB and the presence of moderate or high BF percentages were determined. When determining the predictors of each of the endpoints, logistic regression models were adjusted. The following variables were tested as possible predictors: eating habits, physical activity, sedentary habits, leisure activities, and parents’ BMI. Variables were selected to be included in the model using the Stepwise (Forward) method, together with the Likelihood-Ratio test. For each variable included in the regression model, the adjusted Odds Ratio and the respective 95% confidence interval (CI95%) were also estimated. The quality of the adjustment of the logistic regression models was assessed by determining the area under the Receiver Operating Characteristic curve (AUC) and its sensitivity and specificity. Statistical analyses were conducted using SPSS 19.0*®*, at a 5% significance level for hypothesis-testing.

## Results

Of the 129 adolescents observed between September 2006 and October 2007, 57 (44.2%) were from a rural environment, and 72 (55.8%) were from an urban environment. Adolescents from the rural environment attended a public school, and those from the urban environment attended a private school. The school grade distribution from Year 4 to Year 8 was 10.1%, 31.8%, 29.5%, 27.1% and 1.6%, respectively. A total of 41.9% were boys, and 58.1% were girls; 37.2% were 10 years old, 23.3% were 11, and the remaining 39.5% were 12.

With regards to eating habits (Table 2), the vast majority had breakfast every day, 44.5% drank chocolate milk or coffee and ate cakes/cookies on a daily basis, whereas only 20.3% drank plain milk or ate yogurt. A total of 72.9% of the participants had soup every day. More than half never ate salads and/or boiled vegetables, or ate them four times a week or less. With regard to eating fish and/or meat, 66.7% consumed fish or meat every day, and two children did not even include meat or fish in their diet. Furthermore, 3.9% did not eat fruit, 13.2% ate fruit four times a week or less, and 72.9% of the children ate fruit every day. An excessive amount of high-calorie foods was eaten on a daily basis (candies, desserts, chocolates, pizzas, hamburgers, or ice cream), accounting for 11.6% of the study sample. In the case of high-calorie drinks, 32.6% did not usually drink them, whilst 19.4% drank them every day. Of the 129 adolescents who completed the questionnaire, 47.3% ate four meals a day, 42.6% ate more than four, and 10.1% ate three meals a day.

**Table 2:** Urban versus rural setting: eating habits

Parameter		Urban (*n* = 57)	Rural (*n* = 72)	Total (*n* = 129)	*P*
How often do you have breakfast?	Never	0 (0.0%)	1 (1.4%)	1 (0.8%)	1.000
	4 times a week or less	2 (3.5%)	3 (4.2%)	5 (3.9%)	
	Every day	55 (96.5%)	68 (94.4%)	123 (95.3%)	
What do you have for breakfast?	Coffee and cakes/cookies	24 (42.9%)	33 (45.8%)	57 (44.5%)	0.251
	Plain milk/yogurt	15 (26.8%)	11 (15.3%)	26 (20.3%)	
	Bread with milk/yogurt	17 (30.4%)	28 (38.9%)	45 (35.2%)	
Do you eat soup at lunch or dinner?	Never	2 (3.5%)	3 (4.2%)	5 (3.9%)	0.209
	4 times a week or less	9 (15.8%)	21 (29.2%)	30 (23.3%)	
	1 or more times a day	46 (80.7%)	48 (66.7%)	94 (72.9%)	
Do you eat salad and/or boiled vegetables?	Never	6 (10.5%)	5 (6.9%)	11 (8.5%)	0.191
	4 times a week or less	21 (36.8%)	37 (51.4%)	59 (45.7%)	
	1 or more times a day	30 (52.6%)	29 (40.3%)	59 (45.7%)	
Do you eat fish and/or meat?	Never	0 (0.0%)	2 (2.8%)	2 (1.6%)	0.010
	4 times a week or less	1 (1.8%)	3 (4.2%)	4 (3.1%)	
	More than 4 times a week	10 (17.5%)	27 (37.5%)	37 (28.7%)	
	Everyday	46 (80.7%)	40 (55.6%)	86 (66.7%)	
Do you eat fruit?	Never	2 (3.5%)	3 (4.2%)	5 (3.9%)	<0.001
	4 pieces a week or less	3 (5.3%)	14 (19.4%)	17 (13.2%)	
	1 or more pieces a day	29 (50.9%)	47 (65.3%)	76 (58.9%)	
	3 pieces a day	23 (40.4%)	8 (11.1%)	31 (24.0%)	
Do you eat candies, pizza, hamburgers or ice-cream?	Everyday	3 (5.3%)	12 (16.7%)	15 (11.6%)	0.006
	4 times a week or less	12 (21.1%)	8 (11.1%)	20 (15.5%)	
	More than 4 times a week	17 (29.8%)	35 (48.6%)	52 (40.3%)	
	Rarely	25 (43.9%)	17 (23.6%)	42 (32.6%)	
Do you drink carbonated or non-carbonated high calorie drinks?	Everyday	7 (12.3%)	18 (25.0%)	25 (19.4%)	<0.001
	4 times a week or less	4 (7.0%)	13 (18.1%)	17 (13.2%)	
	More than 4 times a week	16 (28.1%)	29 (40.3%)	45 (34.9%)	
	Rarely	30 (52.6%)	12 (16.7%)	42 (32.6%)	
Number of meals a day	3	4 (7.0%)	9 (12.5%)	13 (10.1%)	0.429
	4	30 (52.6%)	31 (43.1%)	61 (47.3%)	
	More than 4	23 (40.4%)	32 (44.4%)	55 (42.6%)	

Regarding physical activity, 31.7% did not engage in any form of physical activity other than physical education classes at school. Of those who were physically active, 48.8% participated in high-energy activities (≥3 hours a week), and 26.7% did low-energy activities (<2 hours a week). A total of 34.1% went on daily walks of 30 minutes or more. A high percentage of children had sedentary lifestyles habits, as 73.8% spent, on average, two or more hours a day watching television, playing games, or being on a computer/console.

Regarding the nutritional status of the parents, 55.1% of the fathers were overweight, and 10.2% were obese; additionally, 29.1% of the mothers were overweight, and 12.6% were obese. Only 22.1% of the study sample had parents who both had normal weights.

Regarding the nutritional status of the adolescents, 54.9% had a normal weight, 12.4% were overweight, 8.0% were obese, and 8.8% were morbidly obese. At the other end of the scale, 15.9% were underweight, and 12.0% had a BMI between the 10th and 25th percentile.

Normal SSF values were found in 98.2%, and 100% of the adolescents had normal triceps and abdominal skinfold values. With regard to WC, 26.1% had normal values, 28.8% had excessive values, and 40.5% had abdominal obesity. Only 4.5% had low WC values. All children had normal arm and muscle values.

A total of 31.9% of the respondents had a normal BF mass, 9.7% had a high BF, and 24.8% had a very high BF mass. As for lean mass, normal values were obtained in 53.1% of cases. Regarding water weight, 69.0% showed low levels, and 2.7% had an excess amount of water.

- Differences between adolescents in rural and urban environments

It is possible to conclude from the findings presented in Table 1 that adolescents from the two environments differed in terms of baseline characteristics. The urban environment population had more girls (73.7% vs. 5.8%, *p* = 0.001) and a higher age group, as it included more 12-year-olds (56.1% vs. 26.4%), and fewer 10- and 11-year-olds (24.6% vs. 47.2% and 19.3% vs. 26.4%, respectively, *p* = 0.002). Both environments showed similar eating habits (Table 2), in that they had breakfast every day (96.5% in the urban environment and 94.4% in the rural environment, *p* = 1.000) and ate similar foods (*p* = 0.251). In the rural environment, a lower percentage of children drank plain milk or ate yogurt (26.8% in urban vs. 15.6% in rural) and a higher percentage drank chocolate milk or coffee and ate cakes or cookies (42.9% vs. 45.8%). There was very little variation in the consumption of soup and salads and/or boiled vegetables (*p* = 0.209 and *p* = 0.191, respectively). It is important to highlight the fact that a very low percentage of children never ate soup (3.5% in the urban environment vs. 4.2% in the rural environment), or never ate salads or vegetables (10.5% in the urban environment vs. 6.9% in the rural environment). In addition, there was no variation between the sample populations concerning the number of meals eaten daily (*p* = .429).

However, there were some differences in terms of eating habits. A higher percentage of adolescents from the urban environment ate fish or meat (80.7% vs. 55.6%), as well as fruit (40.4% vs. 11.1%). Regarding high-caloric food consumption, 43.9% of adolescents in the urban environment rarely included these in their diet, compared to 23.6% in the rural environment. A higher percentage of adolescents from the rural environment ate high-calorie foods (5.3% vs. 16.7%). The consumption of high-calorie drinks was higher in the rural than in the urban environment. With regard to sedentary behaviour (Table 3), there was little difference in the percentage of adolescents who spent more than 2 hours a day watching television/playing videogames (66.1% in the urban environment vs. 80.0% in the rural environment, *p* = 0.077). As for physical exercise, more students in the rural environment did not engage in activities other than the physical education classes provided at school (14.3% vs. 32.4%), those who went on walks (3.6% vs. 11.3%), or those who went on walks and practiced another activity (16.1% vs. 33.8%). A higher number of adolescents from the urban area were involved in activities other than physical education classes (66.1% vs. 22.5%).

**Table 3:** Urban versus rural setting: physical exercise and a sedentary lifestyle

Parameter		Urban (*n* = 57)	Rural (*n* = 72)	Total (*n* = 129)	*P*
Time spent watching television and playing video games	2 hours or more a day	37 (66.1%)	56 (80.0%)	93 (73.8%)	0.077
	Less than 2 hours a day	19 (33.9%)	14 (20.0%)	33 (26.2%)	
Physical activity besides Physical Education classes	None	8 (14.3%)	23 (32.4%)	31 (24.4%)	<0.001
	Only walking	2 (3.6%)	8 (11.3%)	10 (7.9%)	
	One physical activity	37 (66.1%)	16 (22.5%)	53 (41.7%)	
	Walking + another activity	9 (16.1%)	24 (33.8%)	33 (26.0%)	
If you do another physical activity, how much time do you spend doing it every week	Less than 2 hours a week	14 (30.4%)	9 (22.5%)	23 (26.7%)	0.255
	2 hours a week	8 (17.4%)	13 (32.5%)	21 (24.4%)	
	3 h or more a week	24 (52.2%)	18 (45.0%)	42 (48.8%)	

With regards to the parents (Table 4), there were no statistically significant differences regarding paternal BMI. However, differences were found in mothers’ BMI (*p* < 0.001), with a mean of 23.0 and 26.6 in the urban and rural environments, respectively. Upon further examination of the weight categories, there were also differences between the two environments (*p* = 0.003), with higher percentages of OWOB in the rural environment than in the urban one: 13.9% vs. 37.3% and 5.6% vs. 16.4%, respectively. The percentage of mothers who had a normal weight was higher in the urban environment: 80.6% vs. 46.3%.

**Table 4:** Urban versus rural setting: parent’s body mass index

Parameter		Urban (*n* = 57)	Rural (*n* = 72)	Total (*n* = 129)	*p*
Father	Mean ± Std Dev.	25.6 ± 2.8	26.8 ± 3.4	26.3 ± 3.2	0.081
	Normal	14 (41.2%)	20 (31.3%)	34 (34.7%)	0.443
	Overweight	18 (52.9%)	36 (56.3%)	54 (55.1%)	
	Obese	2 (5.9%)	8 (12.5%)	10 (10.2%)	
Mother	Mean ± Std Dev.	23.0 ± 3.7	26.6 ± 5.0	25.3 ± 4.9	<0.001
	Normal	29 (80.6%)	31 (46.3%)	60 (58.3%)	0.003
	Overweight	5 (13.9%)	25 (37.3%)	30 (29.1%)	
	Obese	2 (5.6%)	11 (16.4%)	13 (12.6%)	
At least one obese parent	No	29 (87.9%)	44 (69.8%)	73 (76.0%)	0.049
	Yes	4 (12.1%)	19 (30.2%)	23 (24.0%)	

Regarding anthropometric parameters at birth, there were no statistically significant differences in the means for weight and length between the two environments: *p* = 0.204 and *p* = 0.375, respectively. All children who participated in the study were full-term births.

With relation to the distribution of adolescents based on BMI, there were no statistically significant differences, *p* = 0.456 (Table 5). The percentages of adolescents who were underweight, normal weight, overweight, obese, or morbidly obese were 14.3%, 52.4%, 16.7%, 11.9% and 4.8% in the urban environment, respectively, and 16.9%, 56.3%, 9.9%, 5.6% and 11.3% in the rural environment, respectively.

**Table 5:** Urban versus rural setting: child’s nutritional status

Parameter		Urban (*n* = 57)	Rural (*n* = 72)	Total (*n* = 129)	*p*
Body Mass Index	Underweight	6 (14.3%)	12 (16.9%)	18 (15.9%)	0.456
	Normal	22 (52.4%)	40 (56.3%)	62 (54.9%)	
	Overweight	7 (16.7%)	7 (9.9%)	14 (12.4%)	
	Obese	5 (11.9%)	4 (5.6%)	9 (8.0%)	
	Morbidly obese	2 (4.8%)	8 (11.3%)	10 (8.8%)	
Waist circumference	Low	1 (2.4%)	4 (5.7%)	5 (4.5%)	0.675
	Normal	9 (22.0%)	20 (28.6%)	29 (26.1%)	
	High	12 (29.3%)	20 (28.6%)	32 (28.8%)	
	Obese	19 (46.3%)	26 (37.1%)	45 (40.5%)	
Body fat	Low/Normal	11 (26.2%)	29 (40.8%)	40 (35.4%)	0.285
	Moderate	15 (35.7%)	19 (26.8%)	34 (30.1%)	
	High	3 (7.1%)	8 (11.3%)	11 (9.7%)	
	Very High	13 (31.0%)	15 (21.1%)	28 (24.8%)	
Water Weight	Low	32 (76.2%)	46 (64.8%)	78 (69.0%)	0.553
	Normal	9 (21.4%)	23 (32.4%)	32 (28.3%)	
	High	1 (2.4%)	2 (2.8%)	3 (2.7%)	
Lean Mass	Low	31 (73.8%)	22 (31.0%)	53 (46.9%)	<0.001
	Normal	11 (26.2%)	49 (69.0%)	60 (53.1%)	
	High	0 (0.0%)	0 (0.0%)	0 (0.0%)	

Further analysis of skinfold measurements showed that there was no difference in the distribution of adolescents according to the standard SSF values between the two environments, *p* = 0.529. There were also no differences regarding the standard WC values, *p* = 0.675. Nevertheless, abdominal obesity was expressed more in both groups, with 46.3% in the urban environment vs. 37.1% in the rural environment. An increased abdominal circumference was evident in 29.3% of urban environment cases, and in 28.6% of rural adolescents. The lowest percentage was found in the low circumference category, with 2.4% in the urban environment, and 5.7% in the rural environment. With regard to body composition, there were no statistically significant differences in target BF (*p* = 0.499) or water weight (*p* = 0.553). Regarding target lean body mass, children from rural environments had higher lean body masses, while it was more common to find children with a low lean body mass in the urban environment, *p* < 0.001 (Table 5).

b) Predictors of overweight/obesity

The adolescents’ environment was analysed to determine whether it could be a risk factor for OWOB. Firstly, a univariate analysis between OWOB and each of the following parameters was carried out: female gender, age equal to 12 years, a rural environment, having breakfast on a daily basis, breakfast comprising chocolate milk or coffee with cakes or cookies, consumption of soup at least once a day, consumption of vegetables at least once a day, consumption of fish and/or meat on a daily basis, consumption of high-calorie foods four times a week or less, non-consumption of high-calorie drinks, consumption of only three main meals a day, daily walks of at least 30 minutes, daily walks and high-energy activities, sedentary leisure activities for at least 2 hours a day (watching television/playing video games), at least one obese parent, an obese father, and an obese mother. The associations between OWOB and each of these factors are presented in Table 6.

**Table 6:** Association between overweight/ obesity and eating habits; physical/leisure activities and parent’s body mass index

% of overweight and obese children in each category		Body mass index				*P*	OR	95% CI	
		Low/Normal		High/Obese				LL	UL
Gender	Male	(34/49)	69.4%	(15/49)	30.6%	0.773	Reference		
	Female	(46/64)	71.9%	(18/64)	28.1%		0.89	0.39	2.01
Age (years)	10/11	(58/76)	76.3%	(18/76)	23.7%	0.064	Reference		
	12	(22/37)	59.5%	(15/37)	40.5%		2.20	0.95	5.10
Setting	Urban	(28/42)	66.7%	(14/42)	33.3%	0.458	Reference		
	Rural	(52/71)	73.2%	(19/71)	26.8%		0.73	0.32	1.68
Has breakfast everyday	No	(5/6)	83.3%	(1/6)	16.7%	0.669	Reference		
	Yes	(75/107)	70.1%	(32/107)	29.9%		2.13	0.24	19.00
Chocolate milk or coffee, cakes/cookies	No	(47/64)	73.4%	(17/64)	26.6%	0.437	Reference		
	Yes	(32/48)	66.7%	(16/48)	33.3%		1.38	0.61	3.13
Soup every day	No	(22/31)	71.0%	(9/31)	29.0%	0.980	Reference		
	Yes	(58/82)	70.7%	(24/82)	29.3%		1.01	0.41	2.51
Vegetables and/or salads > =Once a day	No	(45/60)	75.0%	(15/60)	25.0%	0.296	Reference		
	Yes	(35/53)	66.0%	(18/53)	34.0%		1.54	0.68	3.49
Fish and/or meat everyday	No	(29/39)	74.4%	(10/39)	25.6%	0.545	Reference		
	Yes	(51/74)	68.9%	(23/74)	31.1%		1.31	0.55	3.13
1 or more pieces of fruit a day	No	(17/21)	81.0%	(4/21)	19.0%	0.257	Reference		
	Yes	(63/92)	68.5%	(29/92)	31.5%		1.96	0.60	6.33
Number of candies, pizzas, hamburgers or chocolate eaten a week	> = 4	(26/33)	78.8%	(7/33)	21.2%	0.230	Reference		
	<4	(54/80)	67.5%	(26/80)	32.5%		1.79	0.69	4.66
Never drinks high calorie drinks	No	(59/80)	73.8%	(21/80)	26.3%	0.282	Reference		
	Yes	(21/33)	63.6%	(12/33)	36.4%		1.62	0.68	3.82
Number of meals a day	>3	(73/101)	72.c3%	(28/101)	27.7%	0.328	Reference		
	3	(7/12)	58.3%	(5/12)	41.7%		1.86	0.55	6.36
Walks/High energy activities	No	(60/82)	73.2%	(22/82)	26.8%	0.434	Reference		
	Yes	(19/29)	65.5%	(10/29)	34.5%		1.44	0.58	3.56
≥30 minute walk a day	No	(52/72)	72.2%	(20/72)	27.8%	0.740	Reference		
	Yes	(27/39)	69.2%	(12/39)	30.8%		1.16	0.49	2.71
≥2 hours of television or video games a day	Yes	(60/85)	70.6%	(25/85)	29.4%	0.806	Reference		
	No	(19/26)	73.1%	(7/26)	26.9%		0.88	0.33	2.37
At least one obese parent	No	(59/73)	80.8%	(14/73)	19.2%	0.006	Reference		
	Yes	(12/23)	52.2%	(11/23)	47.8%		3.86	1.42	10.55
Obese father	No	(68/88)	77.3%	(20/88)	22.7%	0.020	Reference		
	Yes	(4/10)	40.0%	(6/10)	60.0%		5.10	1.31	19.87
Obese mother	No	(64/90)	71.1%	(26/90)	28.9%	0.525	Reference		
	Yes	(8/13)	61.5%	(5/13)	38.5%		1.54	0.46	5.14

Based on BMI, 70.8% adolescents were underweight or had normal weight, and 29.2% were overweight or obese. There were no significant correlations between OWOB and gender (*p* = 0.773), residential area (*p* = 0.458), having breakfast daily (*p* = 0.669), having a high-calorie breakfast (chocolate milk or coffee and cakes/cookies) (*p* = 0.437), daily consumption of soup (*p* = 0.980), daily consumption of fish and/or meat (*p* = 0.545), having only 3 meals a day (*p* = 0.328), going for daily walks for over 30 minutes and having another high-energy activity (*p* = 0.434), going for walks for over 30 minutes a day (*p* = 0.740), engaging in sedentary leisure activities for at least 2 hours a day (*p* = 0.806), and having an obese mother (*p* = 0.525).

Only two factors showed a significant association with excess weight: at least one obese parent, and an obese father. Adolescents with one obese parent were 3.86 times more at risk of being overweight than those whose parents were not obese: 19.2% vs. 47.8%, *p* = 0.006. The risk of being overweight in children whose fathers were obese was 5.10 times greater than the risk of those whose fathers were not obese, *p* = 0.020.

A logistic regression model was created to assess whether environment was an independent predictor of OWOB. The conclusion was that living in a rural environment did not predict the adolescent being OWOB: aOR (95% CI) =0.74 (0.25; 2.20), *p* = 0.581. There were other factors that did lead to OWOB, namely, being 12 years of age, eating high-calorie foods four times a week or less, and having at least one obese parent. Therefore, young adolescents who were 12 years old were 3.15 times more likely to be overweight/obese than those who were other ages: aOR (95% CI) =3.15 (1.06; 9.43), *p* = 0.040. The risk of OWOB was 4.74 times greater among adolescents who ate candies less than four times a week when compared to those who ate candies more than four times a week: aOR (95% CI) =4.74 (1.28;17.51). In addition, adolescents with at least one obese parent were 8.29 times more likely to be overweight/obese than other adolescents, aOR (95%CI) =8.29 (2.39; 28.67). The sensitivity and specificity of this model were respectively 68% and 75%, with an AUC (95% CI) of 0.76 (0.65; 0.87).

c) Predictors of moderate or high body fat

As with the OWOB endpoint, the influence of environment on moderate or high BF was also assessed. 40 (35.4%) adolescents had a normal BF, and in 73 cases (64.6%), BF was above normal. The univariate analysis of this endpoint with each of the aforementioned parameters (Table 7) showed that there were no significant correlations between a higher than normal BF and gender (*p* = 0.891), having breakfast every day (*p* = 0.664), having a high-calorie breakfast (*p* = 0.773), consumption of vegetables and/or salads at least once a day (*p* = 0.764), consumption of high-calorie foods less than four times a week (*p* = 0.316), having only three main meals a day (*p* = 0.340), engaging in sedentary leisure activities for at least 2 hours a day (*p* = 0.594), and maternal obesity (*p* = 0.766).

**Table 7:** Association between moderate high body fat and eating habits; physical/leisure activities and parent’s body mass index

% of Moderate or High Body Fat in each category		Body Fat				*p*	OR	95% CI	
		Normal		Moderate/High				LL	UL
Gender	Male	(17/49)	34.7%	(32/49)	65.3%	0.891	Reference		
	Female	(23/64)	35.9%	(41/64)	64.1%		0.95	0.44	2.06
Age (years)	10/11	(32/76)	42.1%	(44/76)	57.9%	0.033	Reference		
	12	(8/37)	21.6%	(29/37)	78.4%		2.64	1.07	6.52
Setting	Urban	(11/42)	26.2%	(31/42)	73.8%	0.115	Reference		
	Rural	(29/71)	40.8%	(42/71)	59.2%		0.51	0.22	1.18
Has breakfast everyday	No	(3/6)	50.0%	(3/6)	50.0%	0.664	Reference		
	Yes	(37/107)	34.6%	(70/107)	65.4%		1.89	0.36	9.84
Chocolate milk or coffee, cakes/cookies	No	(22/64)	34.3%	(42/64)	65.6%	0.733	Reference		
	Yes	(18/48)	37.5%	(30/48)	62.5%		0.87	0.40	1.90
Soup every day	No	(14/31)	45.2%	(17/31)	54.8%	0.182	Reference		
	Yes	(26/82)	31.7%	(56/82)	68.3%		1.77	0.76	4.14
Vegetables and/or salads > =Once a day	No	(22/60)	36.7%	(38/60)	63.3%	0.764	Reference		
	Yes	(18/53)	34.0%	(35/53)	66.0%		1.13	0.52	2.44
Fish and/or meat everyday	No	(17/39)	43.6%	(22/39)	56.4%	0.186	Reference		
	Yes	(23/74)	31.1%	(51/74)	68.9%		1.71	0.77	3.82
1 or more pieces of fruit a day	No	(10/21)	47.6%	(11/21)	52.4%	0.194	Reference		
	Yes	(30/92)	32.6%	(62/92)	67.4%		1.88	0.72	4.91
Number of candies, pizzas, hamburgers or chocolate eaten a week	> = 4	(14/33)	42.4%	(19/33)	57.6%	0.316	Reference		
	<4	(26/80)	32.5%	(54/80)	67.5%		1.53	0.67	3.52
Never drinks high calorie drinks	No	(32/80)	40.0%	(48/80)	60.0%	0.111	Reference		
	Yes	(8/33)	24.2%	(25/33)	75.8%		2.08	0.84	5.19
Number of meals a day	>3	(34/101)	33.7%	(67/101)	66.3%	0.340	Reference		
	3	(6/12)	50.0%	(6/12)	50.0%		0.51	0.15	1.69
Walks/High energy activities	No	(22/82)	26.8%	(60/82)	73.2%	0.002	Reference		
	Yes	(17/29)	58.6%	(12/29)	41.4%		0,26	0,11	0,63
≥30 minute walk a day	No	(19/72)	26.4%	(53/72)	73.6%	0.009	Reference		
	Yes	(20/39)	51.3%	(19/39)	48.7%		0.34	0.15	0.77
≥2 hours of television or video games a day	Yes	(31/85)	36.5%	(54/85)	63.5%	0.594	Reference		
	No	(8/26)	30.8%	(18/26)	69.2%		1.29	0.50	3.32
At least one obese parent	No	(31/73)	42.5%	(42/73)	57.5%	0.073	Reference		
	Yes	(5/23)	21.7%	(18/23)	78.3%		2.66	0.89	7.94
Obese father	No	(35/88)	39.8%	(53/88)	60.2%	0.087	Reference		
	Yes	(1/10)	10.0%	(9/10)	90.0%		5.94	0.72	49.00
Obese mother	No	(33/90)	36.7%	(57/90)	63.3%	0.766	Reference		
	Yes	(4/13)	30.8%	(9/13)	69.2%		1.30	0.37	4.56

Only three parameters showed a significant relationship with high BF: being 12 years old, going for walks and doing high-energy physical activities, and going for walks for at least 30 minutes a day. Those who were twelve year old showed a greater percentage of increased BF than those who were 10 and 11 years old (78.4% vs. 57.9% *p* = 0.033), and they were 2.64 times more at risk of having increased BF. Those who walked for at least 30 minutes a day were 66.0% less likely to develop moderate or high BF than those who did not. Adolescents who were involved in high-energy physical activities in addition to school-based physical education were less likely to have excess BF (74.0%).

The logistic regression analysis showed that rural environment was not an independent predictor of increased BF in adolescents between the ages of 10 and 12: aOR (95%CI) =0.88 (0.35; 2.21), *p* = 0.790. However, being 12 years old, going on walks, and engaging in a high-energy physical activity were predictors of gaining a moderate to high BF.

Twelve-year-olds were 3.12 times more likely to have moderate or high BF than those who were 10 or 11 years old: aOR (95% CI) =3.12 (1.14; 8.52), *p* = 0.027. Adolescents who went on walks and engaged in a high-energy physical activity were 77% less likely to have moderate or high BF: aOR (95% CI) =0.23 (0.09; 0.59), *p* = 0.003. The sensitivity and specificity of this model were 83% and 44%, respectively, with an AUC (95% CI) of 0.70 (0.60; 0.80).

## Discussion

Considering the “healthy” eating habits of children/adolescents, there are some findings from this study that are worth mentioning.

- Analysis of eating habits

- Questionnaire on eating habits: a balanced breakfast should be encouraged, as it is the first meal of the day. Milk and other dairy products, fruit, bread with butter or jam, and freshly squeezed fruit juice are all recommended, and animal fats, chocolate milk, milk with added sugar, cakes, and pastries should be avoided. The vast majority of children/adolescents ate breakfast every day, although they did not always eat the most appropriate foods. Only 35.2% drank milk, ate yogurt or bread with butter/jam/cheese, and did not eat candies or drink chocolate beverages. Almost half the sample (44.5%) drank chocolate milk or coffee with a high sugar content and ate cakes and cookies, which is high in saturated fat and sugars, and is therefore not a healthy option. Parents also made the mistake of adding chocolate to their child’s milk during the first years of children’s lives, fearing that if they did not, their child would not drink it. This, of course, later became a habit. Parents also preferred to give their children cakes and biscuits, perhaps due to the lack of fresh bread in the home, or of not wanting to give them day-old bread. Furthermore, the presence of appealing advertising promotes the consumption of unhealthy foods.

In this study, the majority of children (72.9%) ate soup at least once a day, and 45.7% ate salads and boiled vegetables every day. However, the consumption of these foods does not necessarily help prevent obesity. Meat and fish play an important role in body growth and development, and there was a difference between the two groups regarding the frequency with which these foods were consumed, *p* = 0.010. In the urban environment, meat and fish were eaten more regularly during the week. Studies have shown that children and adolescents, especially those from urban areas and those with greater economic resources, consume excessive amounts of protein, particularly meat [11], as confirmed in this study.

A total of 82.9% of the adolescents ate fruit on a daily basis, and of these, 24% ate three pieces of fruit a day, and 58.9% ate one or two pieces a day. This study showed that more fruit is eaten in urban environments, with approximately 40% of adolescents eating three pieces of fruit a day, compared to 11.1% in the rural environment. Different varieties of fruit are also more abundant year-round in urban environments, as is buying power. This contrasts with the rural environment, where fruit availability is seasonal and thus less varied.

In general, an excessively high amount of high-calorie foods were consumed, although the amount was greater in the rural environment, *p* = 0.006. We believe that parents and adolescents from urban environments have greater food awareness, due to the information available, which may explain these results. In addition, the fact that more fruit was eaten in the urban area reduced the consumption of high-calorie foods in that environment, especially snacks or treats after meals. In the rural environment, there is also a deep-rooted habit, particularly among grandparents, of using food, especially treats, to reward children.

Regarding the consumption of carbonated or non-carbonated drinks, this study showed that the majority of adolescents do not drink soft drinks or drink them four or less times a week. The percentage of adolescents who do not drink soft drinks was higher in the urban than in the rural environment (52.6% vs. 16.7%). We believe that the reasons responsible for this finding are the same as those that motivate parents from the rural environment to give their children high-calorie foods.

Despite the differences between the rural and urban environments with regard to eating habits, it was shown that adolescents who were overweight/obese do not have different eating habits from those who have a normal weight [11].

- Physical Activity: There is a reduction in energy expenditure in the aetiology of obesity that is linked to a decrease in, or a lack of physical activity. Physical activity increases an individual’s energy expenditure, reduces body fat, and contributes to adequate growth, bone development, the development of cardiorespiratory resistance, and greater strength, agility and flexibility [17, 18].

Excessive eating, idleness, advertisements in the media, and sedentary behaviour all contributed significantly to the increase in the incidence of obesity. Sedentary behaviour goes hand in hand with development, with remote controls, lifts, computers, consoles, videogames, cars, and the Internet all contributing to a more sedentary lifestyle. In addition, increasingly in larger cities, the lack of space to practice sports, the increase in violence, the lack of time parents spend with their children, and increased workloads at school also contribute to the child or adolescent becoming more sedentary.

In this sample, 24.4% of adolescents did not practice any physical activity other than their physical education classes at school (normally three hours a week), with the percentages of non-extracurricular physical activity being higher in the rural environment (32.4% in urban vs. 14.3% in rural, *p* = 0.001), probably due to the fact that less sporting opportunities are available in rural environments.

It was also shown that 33.9% of adolescents regularly walked at least 30 minutes a day, with an incidence of 45.1% and 19.6% in the rural and urban environments, respectively. In the rural environment, perhaps due to the lower security concerns, many children walk to school, go on walks, visit their grandparents every day, and participate in church activities. On the other hand, these same children do not have as much access to extracurricular sports. In the city, the ability to commute more easily facilitates involvement in sports, and parents in these environments may have a higher standard of living. Overall, the percentage of adolescents in the urban environment who were involved in a sports activity was 82.1%, compared to 56.3% in the rural environment. By relating physical activity, energy expenditure, and obesity rates in the adolescents observed, it is possible to conclude that medium-to-low-energy expenditure is an independent predictor of obesity, which clearly indicates that local authorities and schools should aim to provide more physical activity opportunities for young people.

Sedentary Behaviour: Sedentary behaviour leads to a lack of physical activity and subsequently a reduction in energy expenditure, which is linked to obesity. According to Ekelund (Ekelund U, 1969), watching television is a positive predictor for increased body fat [19]. In our study, 73.8% spent two or more hours watching television and playing videogames on the computer or on consoles. Sedentary behaviour was greater during the weekend, as young people do not go to school then, and have more free time. There was no difference in sedentary behaviour between the two environments, and therefore it was not an independent predictor of obesity. It is also possible to state that, although there was no conclusive evidence linking physical inactivity to the obesity pandemic, longitudinal studies seem to show a weak association between the two [20].

b) Family history

Over 400 genes or chromosomal regions have been identified and implicated in obesity. Intra-family risks of obesity are due to a common genetic predisposition, triggered by a facilitating obesogenic environment. This crossover, linked to environmental behaviour, creates a strong association between parental and offspring obesity, thus directly correlating the risk of obesity to the degree of parental obesity [21, 22]. In the Portuguese population, a study carried out from 1995 to 1998 showed that 49.6% of parents of obese children were also obese. In another study, which took place between 2003 and 2005, 53.6% of obese children had obese parents [23].

In this study, 55.1% of the fathers and 29.1% of the mothers were overweight, and 10.3% of the fathers and 12.6% of the mothers were obese. When the two environments were compared, there was a greater link between having overweight/obese mothers in the rural, rather than in the urban environment, as well as having a greater number of obese fathers. Thus, having at least one obese parent was an independent predictor of obesity, confirming the notion that sharing the same genetic environment is a determining factor for obesity. This study also showed that 24% had at least one obese parent, with this relationship again being more frequent in the rural environment, 12.1% in urban vs. 30.2% in rural, *p* = 0.049 (Table 4). When adolescents with at least one obese parent were compared to those who did not have obese parents (Table 8), it was observed that the former consumed high-calorie foods, *p* = 0.025 and drinks, *p* = 0.039 more frequently. Additionally, the same group of children with at least one obese parent consumed more meals a day, *p* = 0.030, which mainly consisted of high-calorie foods and less fruit, *p* = 0.047.

**Table 8:** Comparison between parents who are not obese and obese parents

Characteristics		Parents who are not obese	Obese parents	Total	*p*
Female		43 (58.9%)	9 (39.1%)	52 (54.2%)	0.097
Age (years)	10	28 (38.4%)	13 (56.5%)	41 (42.7%)	0.268
	11	18 (24.7%)	5 (21.7%)	23 (24.0%)	
	12	27 (37.0%)	5 (21.7%)	32 (33.3%)	
Setting	Urban	29 (39.7%)	4 (17.4%)	33 (34.4%)	0.049
	Rural	44 (60.3%)	19 (82.6%)	63 (65.6%)	
Number of times breakfast is eaten	Never	0 (0.0%)	1 (4.3%)	1 (1.0%)	0.169
	4 times or less	4 (5.5%)	0 (0.0%)	4 (4.2%)	
	Every day	69 (94.5%)	22 (95.7%)	91 (94.8%)	
What is eaten or drunk for breakfast	Coffee and cake/cookies	28 (38.9%)	9 (39.1%)	37 (38.9%)	0.929
	Plain milk/yogurt	15 (20.8%)	4 (17.4%)	19 (20.0%)	
	Bread with milk/yogurt	29 (40.3%)	10 (43.5%)	39 (41.1%)	
Eating soup at lunch or dinner	Never	2 (2.7%)	2 (8.7%)	4 (4.2%)	0.512
	4 times a week or less	20 (27.4%)	6 (26.1%)	26 (27.1%)	
	1 or more times a day	51 (69.9%)	15 (65.2%)	66 (68.8%)	
Eating salads and/or boiled vegetables	Never	3 (4.1%)	3 (13.0%)	6 (6.3%)	0.112
	4 times a week or less	33 (45.2%)	13 (56.5%)	46 (47.9%)	
	1 or more times a day	37 (50.7%)	7 (30.4%)	44 (45.8%)	
Eating fish and/or meat	Never	1 (1.4%)	1 (4.3%)	2 (2.1%)	0.134
	4 times a week or less	2 (2.7%)	2 (8.7%)	4 (4.2%)	
	>4 times a week	26 (35.6%)	3 (13.0%)	29 (30.2%)	
	Everyday	44 (60.3%)	17 (73.9%)	61 (63.5%)	
Eating fruit	4 pieces a week or less	14 (19.2%)	4 (17.4%)	18 (18.8%)	0.047
	1 or 2 pieces a day	39 (53.4%)	18 (78.3%)	57 (59.4%)	
	3 pieces a day	20 (27.4%)	1 (4.3%)	21 (21.9%)	
Eating candies, pizzas, hamburgers or ice-cream	Everyday	6 (8.2%)	7 (30.4%)	13 (13.5%)	0.025
	4 times a week or more	14 (19.2%)	4 (17.4%)	18 (18.8%)	
	<4 times a week	53 (72.6%)	12 (52.2%)	65 (67.7%)	
Drinks high calorie drinks	4 times a week or more	21 (28.8%)	12 (52.2%)	33 (34.4%)	0.039
	<4 times a week	52 (71.2%)	11 (47.8%)	63 (65.6%)	
Number of meals a day	3	9 (12.3%)	2 (8.7%)	11 (11.5%)	0.030
	4	39 (53.4%)	6 (26.1%)	45 (46.9%)	
	More than 4	25 (34.2%)	15 (65.2%)	40 (41.7%)	
Time spent watching TV and playing video games	2 hours or more a day	57 (80.3%)	15 (65.2%)	72 (76.6%)	0.138
	Less than 2 hours a day	14 (19.7%)	8 (34.8%)	22 (23.4%)	
Physical activity besides Physical Education classes	None	19 (26.4%	4 (17.4%)	23 (24.2%)	0.280
	Only walking	6 (8.3%)	4 (17.4%)	10 (10.5%)	
	Only another activity	25 (34.7%)	11 (47.8%)	36 (37.9%)	
	Walking + another Activity.	22 (30.6%)	4 (17.4%)	26 (27.4%)	
Daily walks of ≥30 minutes	No	44 (61.1%)	15 (65.2%)	59 (62.1%)	0.724
	Yes	28 (38.9%)	8 (34.8%)	36 (37.9%)	

Birth anthropometric parameters: low birth weight is related to a greater risk of adult obesity, and macrosomia to a higher risk of developing diabetes and obesity in the future [24, 25]. In this sample, all adolescents were born full-term; 93.5% were born with weights between the 25th and 50th percentile, 4.2% had high birth weights, and 2.3% had low birth weights. All the newborns with high or low birth weights for their gestational age were from the rural environment. In this study, anthropometry was not an independent predictor of OWOB.

c) Clinical assessment

- Nutritional status and body composition: BMI is also a sensitive indicator of OWOB at a paediatric age [26]. In our study, 12.4% of the adolescents were overweight, 8.0% were obese, 8.8% were morbidly obese, and about half had normal weight.

Intra-abdominal or visceral fat is metabolically active and is responsible for atherogenic dyslipidemia, hyperinsulinemia, hypertension, and metabolic syndrome in adulthood [27–30]. The results of this study showed that 26.1% of adolescents had normal values for their age and gender, 28.8% were between P75 and P95, and 40.5% were above P95 (Table 5). All adolescents who were overweight/obese had a WC higher than P95.

**Fig. 1:**
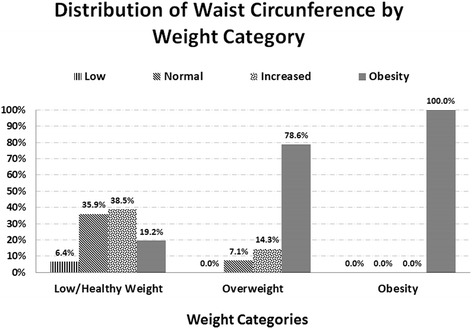
Distribution of waist circumference by weight category

**Fig. 2:**
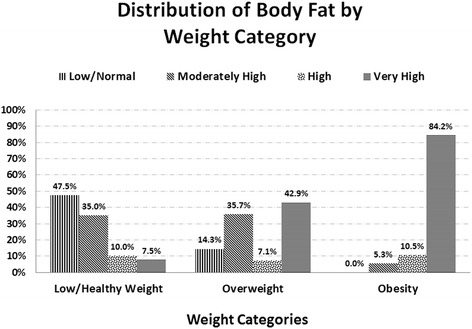
Distribution of body fat by weight category

With regard to their BF percentage, 24.8% of adolescents had very high values, 9.7% had high values, 30.1% had moderate values, and 31.9% had normal values. In Figs. 1 and 2, it is possible to observe that there is a concordance both between being overweight/obese and increased WC or abdominal obesity, and between being overweight/obese and moderate or high BF. Of those adolescents who were overweight, 14.3% had increased WC, and 78.6% were obese, while the obese adolescents all had abdominal obesity. In addition, of those who were overweight and obese, 7.1% and 10.5%, respectively had a high percentage of BF, and 42.9% and 84.2%, respectively had a very high BF percentage.

The findings also showed that the amount of lean mass was normal in only about half of the adolescents, and was low in 46.9% of cases. In addition, 69% showed signs of dehydration. These results indicate that excess BF is generally accompanied by lower percentages of lean mass and water weight. Due to increased energy expenditure, appropriate physical activity results in changes in body composition, reductions in BF, and increases in lean mass. This study showed that 48.8% had high energy expenditure. The amount of water consumed by the adolescents was lower than recommended, which could be because they only drank water at lunch and dinner, and tended to forget to hydrate due to various distractions, or simply because they felt that bringing water to school was “not cool”.

When the two environments were compared with regard to these parameters, there were no significantly significant differences in WC, BF, or water weight. However, there were differences in lean mass. The percentage of adolescents with a low lean mass was higher in the urban environment, 73.8% vs. 31%, which was surprising, although can be potentially explained by the higher percentage of girls in this environment.

This study has some limitations, mostly regarding the selection of schools. The rural school is public (just like most Portuguese rural schools), wheras the urban school is private, although it functions with an agreement with the Portuguese Education Ministry, which means that it has public funding, thus allowing less-well-off urban children to attend this school. Therefore we think that the fact that one of the schools is public, whereas the other is private (but with public funding) is not a limiting factor in our study, neither does it compromise our results and conclusions.

Another possible limitation is the fact that we chose only one school from each environment. In Portugal there few children live in rural environments, and therefore often there is only one school for each district. Therefore we chose only one school from a rural Portuguese environment where children live and study, and also only one from the urban environment. These two schools provided us with a representative and reliable sample for our study.

The authors believe that this study’s findings have enriched the scientific literature, and have established the base for future studies. We think that through this study, we have enabled the development of structured interventions to prevent and decrease the rate of obesity in Portuguese adolescents, which is one of the highest rates in European countries and presents a large public health problem.

## Conclusions

Daily walks of at least 30 minutes a day protect adolescents from moderate or high BF, and high energy expenditure in adolescents prevents excess BF, and consequently obesity.

Having at least one obese parent increases a child’s risk of obesity, and as obese parents tend to provide more high-calorie foods and a greater number of meals, there is a greater risk of their children becoming obese.

From the data obtained in this study, which are consistent with the relevant literature, it is possible to conclude that obesity (BMI > P95) is present in 16.8% of the study population, with OWOB in approximately 30%. In addition, all of the obese adolescents had increased BF and a WC higher than the 90th percentile.

The OWOB rates in the rural and urban environments did not show statistically significant differences.

## Additional file

Additional file 1: Eating habits and leisure activities questionnaire. The first section of the questionnaire aimed to quantify the number of meals and their composition. The second section addressed sedentary activities, such as time spent watching television or on a computer, playing videogames, or engaging in other sedentary activities. It also focused on involvement in physical activity other than the physical education classes provided at school, including time spent on these activities in hours per week. (PDF 40 kb)

## Abbreviations

AC: Arm circumference
AMC: Arm muscle circumference
AUC: Area under the receiver operating characteristic curve
BF: Body fat
BIO: Bioelectrical impedance
BMI: Body mass index
95% CI: 95% confidence interval
aOR: Adjusted odds ratio
OWOB: Overweight or obesity
P75: 75th percentile
P85: 85th percentile
P95: 95th percentile
P97: 97th Percentile
SSF: Subscapular skinfold
TSF: Triceps skinfold
WC: Waist circumference
