# Supplementary material for: Overweight and obesity and their associated factors among early adolescence school children in urban and rural Portugal
Source: BMC Nutr. 2017 Feb 20;3:17. doi: 10.1186/s40795-017-0134-6 (PMC7050717; doi:10.1186/s40795-017-0134-6)
Supplement: Supplementary file 1 — Eating habits and leisure activities questionnaire. The first section of the questionnaire aimed to quantify the number of meals and their composition. The second section addressed sedentary activities, such as time spent watching television or on a computer, playing videogames, or engaging in other sedentary activities. It also focused on involvement in physical activity other than the physical education classes provided at school, including time spent on these activities in hours per week. (PDF 40 kb) [file 40795_2017_134_MOESM1_ESM.pdf]

Questionnaire – Evaluation of the Nutritional Status

Name of the student:

Date of birth:

Educational establishment:

School year: **Class:** **Nº:**

Answer as correctly as possible to the following questions. Write the word 'True' on the selected option.

1 – Do it eat breakfast? (select only one answer)

a) every day

b) less then 4 times a week

c) never eat breakfast

2 – Usually I eat and drink at breakfast (select all the correct answers)

a) bread with butter, ham, cheese

b) cakes, cookies or other sweetmeats

c) plain milk or yogurt

d) chocolate milk, cocoa or coffee

3 – Relatively to lunch and dinner (select only one answer)

a) always eat soap at least once a day

b) only eat soap 4 or less times a week

c) never eat soap

Relatively to vegetables and salads (select only one answer)

a) eat salads and/or boiled vegetables every day

b) only eat salads and/or boiled vegetables 4 or less times a week

c) usually never eat salads and/or boiled vegetables

Fish and/or meat (select only one answer)

a) eat fish and/or meat every day

b) eat fish and/or meat at least 4 times a week

c) eat fish and/or meat less then 4 times a week

d) usually don’t eat fish nor meat

4 – Fruit (don’t consider canned fruit) (select only one answer)

a) eat daily 3 or more pieces of fruit

b) eat at least 1 piece of fruit daily

c) eat 4 or less pieces of fruit per week

d) usually never eat fruit

5 – Candies, desserts, chocolates, pizzas, hamburgers, ice creams (select only one answer)

a) every day I eat 1 of the mentioned foods

b) eat at least 4 times a week one of the mentioned foods

c) eat less then 4 times a week one of the mentioned foods

d) usually don’t eat any of the mentioned foods

6 – Soft drinks, capsule juices or tin with or without gas, coca-cola (select only one answer)

a) drink daily one of the mentioned drinks

b) drink more then 4 times a week one of the mentioned drinks

c) drink less then 4 times a week one of the mentioned drinks

d) usually don’t drink any of the mentioned drinks

7 – Number of daily meals (select only one answer)

a) I have more then 4 meals a day

b) usually I have 4 meals a day

c) usually I have 3 meals a day

Leisure time

Sports or cultural activities

1- Do you practice any sport activity, besides the Physical Education you have at school?

Yes No

Which one?

If you do, how many hours a week?

a) 3 or more hours

b) 2 hours

c) less then 2 hours

Do you usually walk more then 30 minutes a day? (select only one answer)

a) Yes

b) No

Daily habits – television or videogames like Game Boy, Playstation, PSP

Daily you watch television and/or play videogames more then 120 minutes (or 2 hours)

Daily you watch television and/or play videogames less then 120 minutes (or 2 hours)
